# Plasminogen activator inhibitor-1, vaspin, and dietary inflammatory index in relation to cardiometabolic health in diabetic women

**DOI:** 10.3389/fnut.2026.1784603

**Published:** 2026-04-10

**Authors:** Sule Kocabas, Nevin Sanlier

**Affiliations:** Department of Nutrition and Dietetics, School of Health Sciences, Ankara Medipol University, Altındaǧ, Ankara, Türkiye

**Keywords:** dietary inflammatory index, energy-adjusted diet inflammatory index, PAI-1, type 2 diabetes mellitus, vaspin

## Abstract

**Background:**

Chronic systemic inflammation is strongly associated with type 2 diabetes mellitus (T2DM) and related cardiometabolic conditions. Adipokines such as plasminogen activator inhibitor-1 (PAI-1) and vaspin are linked to obesity, insulin resistance, and inflammation. This study examines the associations of PAI-1 and vaspin levels with cardiometabolic risk markers and the inflammatory potential of diet in overweight or obese women with and without T2DM.

**Methods:**

Women aged 20–50 were grouped based on diabetes diagnosis. Anthropometric measurements, biochemical data (fasting glucose, insulin, HbA1c, and lipid profile), and adiposity indices (Visceral Adiposity Index, Body Adiposity Index, Lipid Accumulation Product Index, and Conicity Index) were assessed. Nutritional status was analyzed via 3-day dietary records. Dietary Inflammatory Index (DII) and Energy-Adjusted DII (E-DII) scores were computed. PAI-1 and vaspin levels were measured by ELISA.

**Results:**

PAI-1 levels were significantly higher in the T2DM group (*p* < 0.05), whereas vaspin levels did not differ significantly between groups (*p* > 0.05). After adjustment for age, BMI, and waist circumference (Models 3 and 4), DII and E-DII scores were not significantly associated with T2DM status (*p* > 0.05). In the T2DM group, higher E-DII scores were associated with lower intake of fiber, polyunsaturated fat, vitamin E, thiamine, folate, vitamin C, zinc, and magnesium (*p* < 0.05).

**Conclusion:**

Elevated PAI-1 levels were found in women with T2DM, while dietary inflammatory potential was not independently associated with T2DM status after adjustment for adiposity-related factors. Higher E-DII scores were also associated with lower intake of anti-inflammatory nutrients. These findings highlight the importance of accounting for confounding variables in diet-related inflammation research.

## Introduction

Type 2 diabetes mellitus (T2DM) has become a major public health issue due to its rapidly increasing prevalence worldwide and the serious complications it causes. According to global disease burden data, more than 500 million adults worldwide live with diabetes, and T2DM accounts for over 90% of these cases (1). The association between diabetes and cardiovascular disease carries a higher mortality risk for women compared to men. This makes the assessment of cardiometabolic health indicators even more important (2). Cardiometabolic health is defined by the combined assessment of obesity, insulin resistance, dyslipidemia, hypertension, and inflammatory conditions, necessitating a multidisciplinary approach to diabetes management (3). Urbanization, rising obesity rates, and physical inactivity in low- and middle-income countries play significant roles in the global increase in T2DM prevalence (4).

Certain biomarkers are prominent in the assessment of cardiometabolic risk. Plasminogen activator inhibitor-1 (PAI-1), one of the main inhibitors of the fibrinolytic system, is associated with obesity, insulin resistance, and atherosclerotic processes (5). On the other hand, vaspin, a serine protease inhibitor secreted by visceral adipose tissue, is among the new generation of adipokines thought to have positive effects on insulin sensitivity, and its connection to the pathophysiology of diabetes is being increasingly researched (6). These two adipokines stand out as potential biomarkers that could influence the disease course in women with diabetes.

In diabetes management, not only biochemical indicators but also the quality of an individual's diet plays an important role. The effect of nutrition on the inflammatory response has been intensively researched in recent years, and the Dietary Inflammatory Index (DII) is used as a quantitative measurement tool (7). The DII allows for the assessment of an individual's dietary inflammatory potential, and this score has been shown to be associated with cardiometabolic health indicators (8, 9). A cross-sectional study conducted in the Italian population showed higher levels of inflammatory dietary habits in patients with acute myocardial infarction (9). This finding has been similarly confirmed in the United States, where higher DII scores are associated with an increased risk of cardiovascular disease, diabetes, and hypertension (8). However, the interaction of the DII with specific inflammatory and metabolic markers such as PAI-1 and vaspin has not been sufficiently investigated, particularly in the female diabetic population.

This study aims to investigate the relationships between PAI-1 and vaspin levels and DII scores in women with diabetes, thereby revealing the effects of these parameters on cardiometabolic health indicators.

## Methods

### Study population

**Figure 1:**
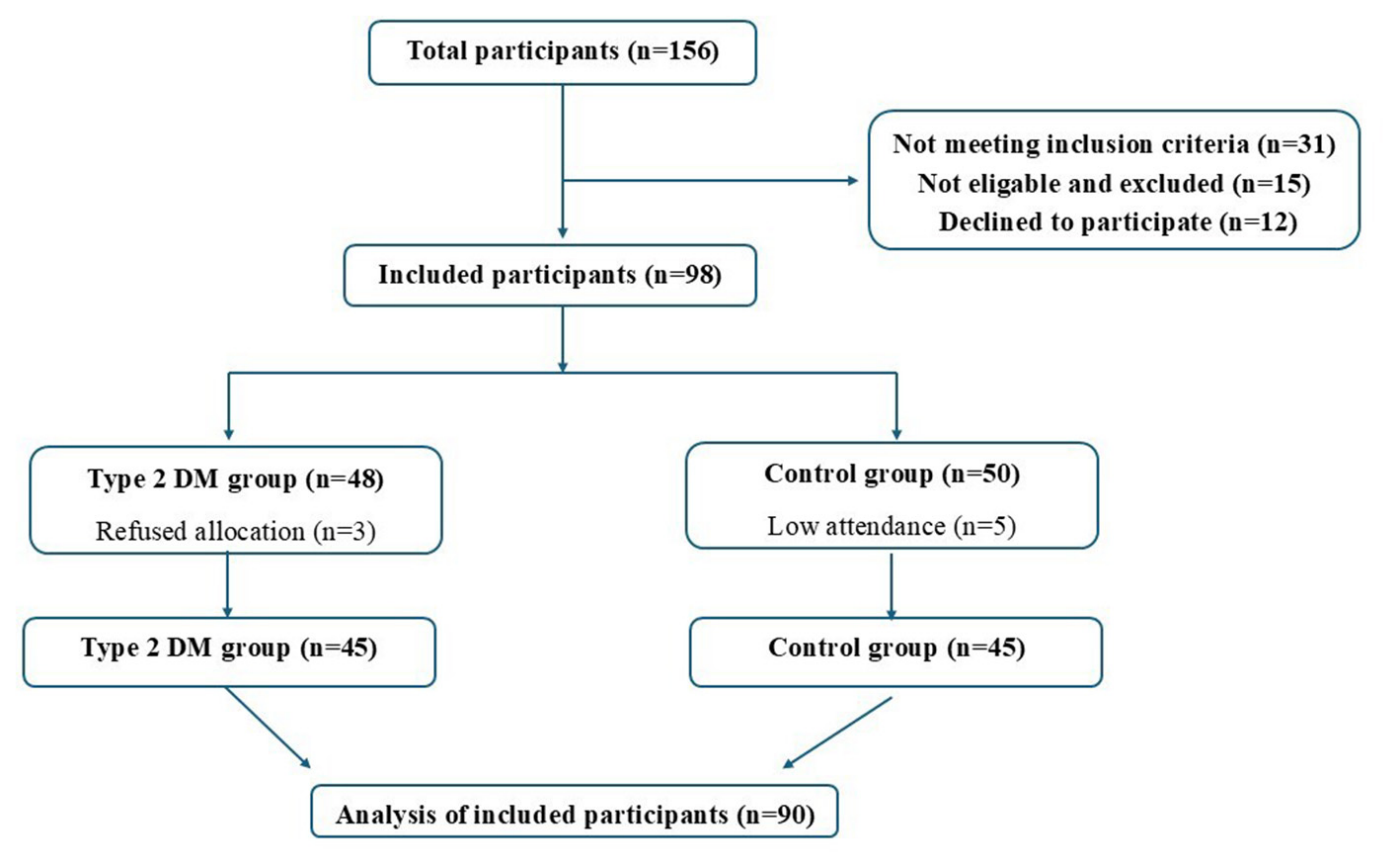
Participant flow chart throughout the study.

This study was conducted between November 2023 and May 2024 with overweight or obese women aged 20–50 who visited the Internal Medicine Outpatient Clinic of a state hospital. The case group consisted of individuals who had been diagnosed with T2DM for at least 1 year, while the control group consisted of individuals with similar characteristics but no diagnosis of T2DM. To determine the sample size, an analysis was performed using the G^*^Power 3.1.9.7 program with α = 0.05, effect size of *d* = 0.73, and power (1–β) of 0.90. According to this calculation, at least 41 women were needed for each group, and the study was completed with a total of 90 women, including 45 with T2DM and 45 in the control group. The study flowchart is shown in Figure 1. The inclusion criteria for both groups were being between 20 and 50 years of age, being overweight or obese, and not being in menopause. Additionally, case group participants had a T2DM diagnosis for at least 1 year. Exclusion criteria included being under 20 or over 50 years of age, having a normal or low body mass index (BMI), having type 1 or gestational diabetes, and having certain other chronic diseases (cardiovascular, liver, and kidney), psychiatric illnesses, eating disorders, cancer, medication use (corticosteroids, etc.), pregnancy, breastfeeding, history of bariatric surgery, or alcohol/substance dependence. Ethical approval for the study was obtained from the university's Health Sciences Non-Interventional Ethics Committee on October 18, 2023 (No. 133). Participants were informed about the purpose and methods of the study and they signed written consent forms indicating their voluntary participation.

### General information

The demographic characteristics of the participants (age, marital status, educational status, employment status, occupational status, and smoking habits) were recorded. Information regarding current medication use, including antidiabetic drugs (oral antidiabetics and/or insulin), lipid-lowering agents, and antihypertensive medications, was obtained from medical records and participant interviews.

### Anthropometric measurements

All anthropometric measurements (body weight, height, and waist, hip, and neck circumferences) of the individuals participating in the study were performed by the researcher. Waist-to-hip ratios, waist-to-height ratios, and BMI values were calculated based on the results of these anthropometric measurement. BMI values were obtained by dividing the individual's body weight (kg) by the square of her height (m^2^; kg/m^2^). Classification was performed according to the guidelines of the World Health Organization (WHO), with BMI values of < 18.5 kg/m^2^ classified as underweight, 18.5–24.9 kg/m^2^ as normal weight, 25.0–29.9 kg/m^2^ as overweight, and ≥30.0 kg/m^2^ as obese (10).

### Adiposity indices

Various index values were calculated as part of the adiposity assessments. The Visceral Adiposity Index (VAI) was applied to assess visceral fat accumulation and predict cardiovascular risks more accurately. For women, VAI scores are calculated using the following formula: {waist circumference / [36.58 + (1.89 × BMI)] × [(TG/0.81) × (1.52/HDL-C)]}. Waist circumference was measured in cm, while triglyceride (TG) and high-density lipoprotein cholesterol (HDL-C) were measured in mmol/L (11).

The Body Adiposity Index (BAI) is calculated as follows to estimate an individual's body fat percentage regardless of ethnic differences: [hip circumference (cm)/height (m)^1.5^]−18. This method is a practical measure that is valid regardless of sex (12).

The Lipid Accumulation Products Index (LAP) is calculated as follows to assess abdominal fat in women: [waist circumference (cm)−58] × TG (mmol/L). This index is used to reflect risks associated with lipid metabolism (13).

The Conicity Index (CI) is a new anthropometric parameter that assesses abdominal adiposity and provides benefits in predicting cardiovascular risks. For women, it is calculated using the following formula: waist circumference (m) / {0.109 × √[body weight (kg)/height (m)]} (14).

### Framingham risk score (FRS)

The FRS was used to determine participants' cardiovascular risk levels. This score is calculated based on age, sex, smoking status, presence of diabetes mellitus, total cholesterol, HDL-C, and systolic and diastolic blood pressure. The FRS provides the percentage risk of developing coronary artery disease within 10 years. In risk classification, < 10% is considered low risk, 10–20% indicates moderate risk, and >20% signifies high risk (15).

### Food consumption

The researcher assessed the participants' general dietary habits and nutritional status. Following blood sampling, bioelectrical impedance analysis of body weight, and anthropometric measurements, a 3-day nonconsecutive dietary recall method was applied over a period of 3 weeks. A photo-illustrated food portion measurement catalog was used to determine food quantities (16). Records were obtained through face-to-face interviews and/or telephone calls. Daily energy and macro- and micronutrient intakes were calculated using the BeBiS 9.1 program.

### Biochemical parameters

Fasting blood samples were collected from participants as part of the routine tests requested by their physicians and analyzed in the biochemistry laboratory of a state hospital. The biochemical findings evaluated included fasting plasma glucose, insulin, HbA1c, Homeostatic Model Assessment for Insulin Resistance (HOMA-IR) scores, TG, total cholesterol (TC), HDL-C, low-density lipoprotein cholesterol (LDL-C), systolic and diastolic blood pressure, and PAI-1 and vaspin levels. Insulin resistance was calculated using the HOMA-IR model [HOMA = fasting glucose (mg/dL) × insulin (μU/mL) / 405]. The Atherogenic Plasma Index was calculated using the ratio of TG to HDL-C. PAI-1 and vaspin levels were obtained from plasma remaining after routine blood collection; no additional samples were taken for this process. Blood samples were centrifuged at 3,000 rpm for 15 minu, and then plasma was transferred to 1.5-mL Eppendorf tubes and stored at −40°C until the day of analysis. Subsequently, PAI-1 and vaspin levels were analyzed by enzyme-linked immunosorbent assay according to the manufacturer's instructions and in duplicate.

Serum plasminogen activator inhibitor-1 (PAI-1) and vaspin levels were measured using commercially available enzyme-linked immunosorbent assay (ELISA) kits according to the manufacturer's instructions. Human PAI-1 concentrations were determined using a Human PAI-1 ELISA kit (SunRed Biological Technology, Shanghai, China; Catalog No: 201-12-1160). The assay sensitivity was 0.05 ng/mL and the measurement range was 0.1–20 ng/mL. Serum vaspin levels were measured using a Human vaspin ELISA kit (SunRed Biological Technology, Shanghai, China; Catalog No: 201-12-0922). The assay sensitivity was 12.371 pg/mL with a detection range of 15–3500 pg/mL. The intra-assay and inter-assay coefficients of variation were < 10 and < 12%, respectively. All assays were performed according to the manufacturer's protocols using duplicate measurements. Absorbance values were read at 450 nm using a microplate reader, and concentrations were calculated from the standard curve generated using known concentrations provided in the kits. Samples exceeding the upper detection limit were appropriately diluted and re-measured.

### Dietary inflammatory index (DII) and energy-adjusted dietary inflammatory index (E-DII)

DII scores are calculated according to the index developed by Shivappa et al. based on 45 food components (17). The average 3-day food intake of the participants was analyzed using the Nutrient Database Programme (BeBiS, Ebispro for Windows, Germany; Turkish Version/BeBiS 8.2). The DII score was calculated based on the consumption amounts of 37 foods and nutrients. Since the BeBIS program does not include the flavan-3-ol, flavon, flavonol, flavonone, anthocyanidin, isoflavone, trans fat, and eugenol contents of foods, they were not included in the DII calculation. The E-DII score was calculated based on consumption per 1,000 kcal, more accurately reflecting the inflammatory potential of the diet by taking into account individual energy intake differences (18). High E-DII scores indicate pro-inflammatory diets, while low scores indicate anti-inflammatory diets (18).

### Statistical analysis

All statistical analyses were performed using IBM SPSS Statistics 27. Findings were summarized with frequency tables and descriptive statistics. For data showing normal distribution, the independent samples *t*-test was applied for comparisons between two groups and analysis of variance testing was applied for comparisons of three or more groups. Pairwise comparisons were performed using the Tukey test for homogeneity of variance and the Tamhane test when homogeneity was not achieved. For data that did not conform to normal distribution, Mann-Whitney U tests and Kruskal-Wallis H tests were used, respectively. The relationships between two qualitative variables were evaluated using the Pearson chi-square test. Correlations between two quantitative variables were analyzed using Pearson or Spearman correlation coefficients, depending on the degree of normality. Logistic regression analysis was used when the dependent variable was categorical, and model fit was evaluated using the Hosmer-Lemeshow test. Significance was accepted at *p* < 0.05 in all analyses.

## Results

This study included a total of 90 female participants, divided into two groups as those diagnosed with T2DM (*n* = 45) and those not diagnosed (*n* = 45). The mean age of the participants was 45.04 ± 0.57 years in the T2DM group and 38.87 ± 0.58 years in the control group (*p* < 0.001). The majority of individuals with T2DM had completed primary school (37.7%), while the majority of those in the control group had completed university or postgraduate studies (46.7%). In the T2DM group, 64.5% of the participants were housewives, compared to 46.7% in the control group. Among individuals with T2DM, 88.9% reported using antidiabetic medication, while 11.1% were not using any antidiabetic treatment. None of the control participants reported antidiabetic medication use. There were no statistically significant differences between the groups in terms of employment status, smoking habits, or daily number of cigarettes (*p* > 0.05). None of the participants consumed alcoholic beverages (Table 1).

**Table 1:** General characteristics of the participants (*n* = 90).

Variables	T2DM group (*n* = 45)		Control group (*n* = 45)		Z^*^/χ^2**^
	*n*	%	*n*	%	*p*
Age (years)					
Mean ± SD	45.0 ± 4.57		38.8 ± 7.58		*Z* = −4.029
Median [IQR]	47.0 [6.5]		39.0 [15.0]		***p*** **<** **0.001**
Marital status					
Single	5	11.1	12	26.7	χ^2^ = 3.554
Married	40	88.9	33	73.3	***p*** **<** **0.05**
Educational status					
Primary school	17	37.7	7	15.5	
Middle school	7	15.6	3	6.7	χ^2^ = 12.767
High school	14	31.1	14	31.1	***p*** **=** **0.005**
University/postgraduate	7	15.6	21	46.7	
Employment status					
Working	14	31.1	18	40.0	χ^2^ = 0.776
Not working	31	68.9	27	60.0	*p* = 0.378
Occupational status					
Civil servant	4	8.9	8	17.8	
Private sector	5	11.1	2	4.4	χ^2^ = 12.042
Self-employed	5	11.1	14	31.1	***p*** **=** **0.034**
Housewife	29	64.5	21	46.7	
Retired	2	4.4	–	–	
Antidiabetic medication use					
Yes	40	88.9	–	–	
No	5	11.1	–	–	
Smoking habits					
Smoker	13	28.9	12	26.7	χ^2^ = 0.055
Non-smoker	32	71.1	33	73.3	*p* = 0.814
Daily cigarette consumption (cigarettes/day)					
< 10	4	30.8	1	8.3	χ^2^ = 1.965
10–15	5	38.4	6	50.0	*p* = 0.374
≥20	4	30.8	5	41.7	

^*^Mann-Whitney U test (Z-table value) statistics were used to compare the measurements of two independent groups for data that did not have normal distribution.

^**^Pearson chi-square cross-tabulations were used to examine relationships between two qualitative variables.

Values with *p* < 0.05 are written in bold.

Waist circumference measurements were higher in the T2DM group (104.61 ± 3.31 cm) than in the control group (99.69 ± 0.89 cm) and the difference was statistically significant (*p* < 0.05). Hip circumference measurements were similar in the T2DM and control groups (*p* > 0.05). The difference in waist-to-hip ratio was statistically significant, with the mean ratio of the T2DM group (0.90 ± 0.08) being higher than that of the control group (0.80 ± 0.06; *p* < 0.05). The waist-to-height ratio also differed significantly between the groups, being higher in the T2DM group (*p* < 0.05). Mean VAI, LAP, CI, and FRS scores were higher in the T2DM group compared to the control group (*p* < 0.05). In the T2DM group, statistically significant differences were found compared to the control group for fasting blood glucose, HbA1c, HOMA-IR score, TG, ratio of TG to HDL-C, systolic blood pressure, diastolic blood pressure, and PAI-1 values (*p* < 0.05 for all). Plasma PAI-1 values in the T2DM group (7.07 ± 0.33 ng/mL) were also higher than those of the control group (3.22 ± 0.36 ng/mL; *p* < 0.05). There were no statistically significant differences between the T2DM and control groups in terms of insulin, total cholesterol, LDL-C, HDL-C, hemoglobin, creatinine, urea, C-reactive protein (CRP), or vaspin (pg/mL) values (*p* > 0.05; Table 2).

**Table 2:** Mean, standard deviation (SD), and median [IQR] values of participants' anthropometric measurements and biochemical findings.

Variables	T2DM group (*n* = 45)		Control group (*n* = 45)		Z^*^/t^**^ p
	Mean ±SD	Median [IQR]	Mean ±SD	Median [IQR]	
Body weight (kg)	83.1 ± 14.35	80.7 [16.6]	84.9 ± 13.31	82.6 [18.3]	
Height (cm)	161.6 ± 5.86	160.0 [9.5]	164.3 ± 6.34	165.0 [8.5]	
BMI (kg/m^2^)	31.8 ± 5.03	30.8 [6.1]	31.4 ± 4.39	30.3 [7.0]	*Z* = −0.190 *p* = 0.850
Visceral adiposity index (VAI)	5.5 ± 2.32	5.2 [2.7]	5.1 ± 4.54	4.0 [3.3]	*Z* = −2.490 ***p*** **=** **0.013**
Body adiposity index (BAI)	580.9 ± 82.56	568.5 [104.4]	577.0 ± 77.12	564.6 [124.9]	*Z* = −0.214 *p* = 0.831
Lipid accumulation products index (LAP)	172.7 ± 90.95	157.8 [98.0]	144.9 ± 114.27	115.6 [68.3]	*Z* = −2.820 ***p*** **=** **0.005**
Conicity index (CI)	1.3 ± 0.11	1.34 [0.1]	1.2 ± 0.08	1.27 [0.1]	*Z* = −3.886 ***p*** **<** **0.001**
Framingham risk score (FRS)	10.2 ± 4.71	10.0 [7.5]	6.7 ± 6.50	6.0 [10.0]	*t* = 2.914 ***p*** **=** **0.005**
Fasting blood glucose (mg/dL)	139.5 ± 60.87	125.5 [42.4]	92.3 ± 12.82	90.7 [9.9]	*Z* = −6.488 ***p*** **<** **0.001**
Insulin (uIU/dL)	12.4 ± 6.57	11.2 [7.2]	13.3 ± 11.56	10.7 [8.3]	*Z* = −0.085 *p* = 0.932
HbA1c (%)	6.7 ± 1.40	6.3 [1.9]	5.4 ± 0.42	5.4 [0.5]	*Z* = −5.307 ***p*** **<** **0.001**
HOMA-IR score	4.3 ± 3.21	3.5 [2.6]	3.1 ± 2.77	2.4 [2.1]	*Z* = −2.522 ***p*** **=** **0.012**
Triglycerides (mg/dL)	143.9 ± 58.54	143.0 [54.5]	135.8 ± 98.01	109.0 [66.5]	*Z* = −2.324 ***p*** **=** **0.020**
Total cholesterol (mg/dL)	207.6 ± 35.57	204.0 [50.5]	202.5 ± 36.96	201.0 [50.0]	*t* = 0.665 *p* = 0.508
LDL-C (mg/dL)	123.7 ± 26.92	123.0 [30.5]	131.3 ± 65.51	124.0 [40.5]	*Z* = −0.343 *p* = 0.732
HDL-C (mg/dL)	54.2 ± 10.96	54.0 [17.0]	57.4 ± 18.77	55.0 [12.5]	*Z* = −0.275 *p* = 0.784
Triglycerides-to-HDL ratio	2.7 ± 1.10	2.7 [1.4]	2.6 ± 2.23	2.0 [1.4]	*Z* = −2.195 ***p*** **=** **0.028**
Systolic blood pressure (mmHg)	124.3 ± 13.35	123.0 [22.0]	116.9 ± 15.46	115.0 [12.0]	*Z* = −2.894 ***p*** **=** **0.004**
Diastolic blood pressure (mmHg)	79.3 ± 7.11	78.0 [9.0]	74.7 ± 8.52	74.0 [10.0]	*t* = 2.806 ***p*** **=** **0.006**
PAI-1 (ng/mL)	7.0 ± 7.33	3.8 [6.1]	3.2 ± 2.36	2.7 [2.6]	*Z* = −2.647 ***p*** **=** **0.008**
Vaspin (pg/mL)	1,190.6 ± 1,133.29	841.4 [1,174.2]	852.4 ± 522.56	687.2 [493.1]	*Z* = −0.512 *p* = 0.608

^*^For data that did not have normal distribution, Mann-Whitney U test (Z-table values) statistics were used to compare measurement values of two independent groups.

^**^For data that had normal distribution, independent sample t-test (t-table values) statistics were used to compare measurement values of two independent groups.

Values with *p* < 0.05 are written in bold.

**Figure 2:**
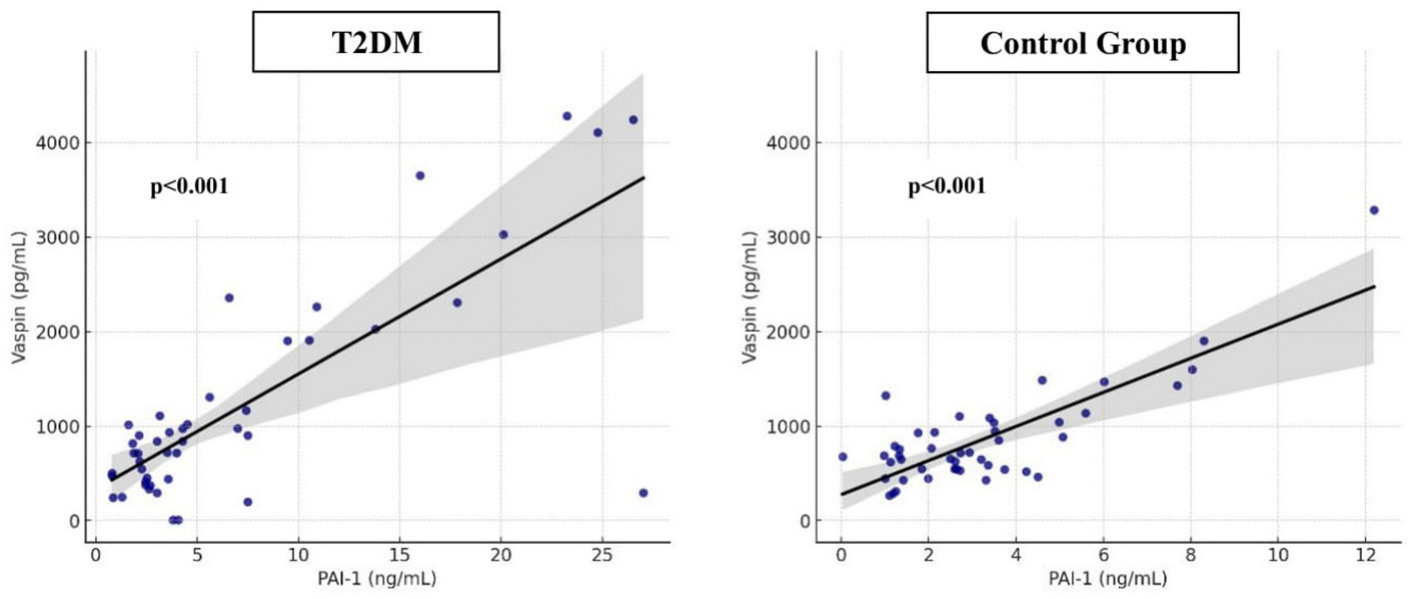
Relationship between PAI-1 and vaspin levels in women with T2DM and the control group. Dots indicate individuals' PAI-1 and vaspin levels. Black lines are linear regression curves showing the relationships. The gray shaded areas indicate the 95% confidence intervals.

The relationships between PAI-1 and vaspin levels in women with T2DM and the women in the control group are shown in Figure 2.

Age, HbA1c, PAI-1, and vaspin levels were identified as significant factors associated with T2DM status. Each one-year increase in age was associated with 16.8% higher odds of T2DM (OR: 1.168; *p* < 0.001). Each unit increase in HbA1c (%) was associated with 7.83-fold higher odds of T2DM (OR: 7.828; *p* < 0.001). Each 1 ng/mL increase in PAI-1 was associated with a 60.8% increase in the odds of T2DM (OR: 1.608; *p* = 0.006), and each 1 pg/mL increase in vaspin was associated with a 0.3% increase in the odds of T2DM (OR: 1.003; *p* = 0.035). The Framingham Risk Score was significantly associated with T2DM status (OR: 1.117; *p* = 0.007). In Models 3 and 4, after adjustment for age, BMI, and waist circumference, DII and E-DII scores were not significantly associated with T2DM status. In Model 3, the DII score showed no statistically significant association with T2DM status (OR: 0.890; 95% CI: 0.790–1.014; *p* = 0.063). Each one-year increase in age was associated with approximately 18% higher odds of T2DM (OR: 1.180; *p* < 0.001), while each 1-cm increase in waist circumference was associated with about 10% higher odds of T2DM (OR: 1.100; *p* = 0.008). Similarly, in Model 4, the E-DII score was not significantly associated with T2DM status (OR: 0.890; 95% CI: 0.770–1.020; *p* = 0.088; Table 3).

**Table 3:** Analysis of some factors affecting T2DM using logistic regression in different models.

Variables	β	Standard error	Wald	df	*p*	OR	95% confidence interval (OR)	
							Min	Max
Model 1								
Age (years)	0.155	0.040	14.941	1	**< 0.001**	1.168	1.080	1.264
HbA1c (%)	2.058	0.571	12.979	1	**< 0.001**	7.828	2.556	23.980
PAI-1 (ng/mL)	0.475	0.173	7.539	1	**0.006**	1.608	1.146	2.256
Vaspin (pg/mL)	0.002	0.001	4.460	1	**0.035**	1.003	1.001	1.005
Constant term	−12.395	3.331	14.012	1	**< 0.001**	0.001		
Model 2								
Framingham risk score	0.111	0.041	7.295	1	**0.007**	1.117	1.031	1.210
Constant term	−1.012	1.131	0.802	1	0.371	0.364		
Model 3								
Age (years)	0.167	0.047	12.750	1	**< 0.001**	1.180	1.070	1.290
BMI	−0.115	0.085	1.830	1	0.176	0.890	0.750	1.060
Waist circumference (cm)	0.096	0.036	7.090	1	**0.008**	1.100	1.020	1.180
DII score	−0.117	0.063	3.460	1	0.063	0.890	0.790	1.014
Constant term	−13.361	3.669	13.27	1	**< 0.001**	0.001		
Model 4								
Age (years)	0.169	0.047	13.080	1	**< 0.001**	1.180	1.070	1.290
BMI	−0.113	0.085	1.750	1	0.186	0.890	0.750	1.060
Waist circumference (cm)	0.096	0.037	6.860	1	**0.009**	1.100	1.020	1.180
E-DII score	−0.121	0.071	2.910	1	0.088	0.890	0.770	1.020
Constant term	−13.367	3.680	13.190	1	**< 0.001**	0.001		

Values with *p* < 0.05 are written in bold.

**Figure 3:**
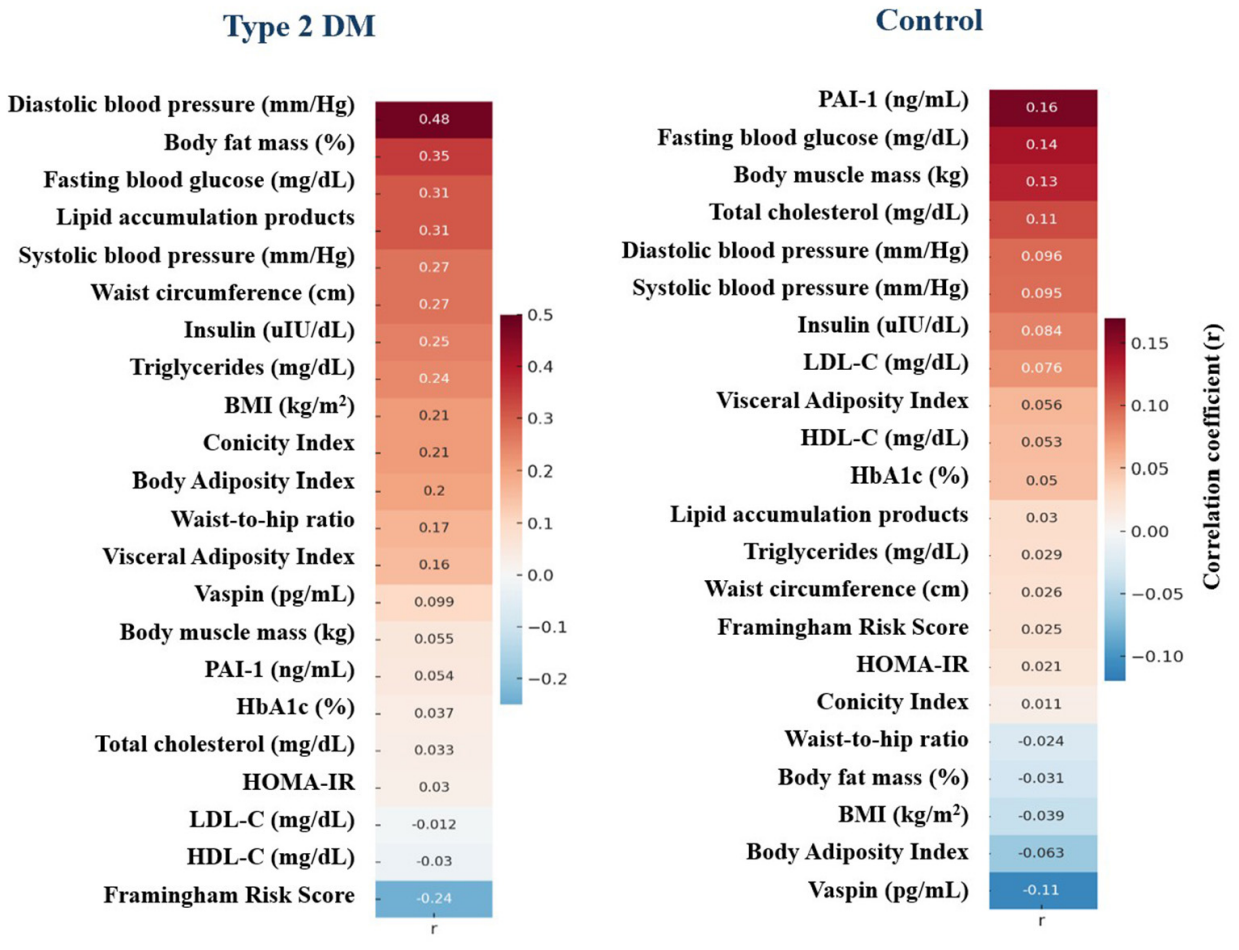
Heat map of correlations between E-DII scores and some biochemical values and anthropometric measurements for the T2DM and control groups. The color scale was created based on the magnitude of the correlation coefficients (*r*), making it easier to observe the direction and strength of the relationships between variables.

The heat map showing the correlations between E-DII scores and some biochemical values and anthropometric measurements of the T2DM and control groups is presented in Figure 3. In the T2DM group, strong positive correlations between E-DII score and diastolic blood pressure (*r* = 0.481), body fat mass (*r* = 0.346), lipid accumulation products (*r* = 0.310), HOMA-IR score (*r* = 0.309), and fasting blood glucose (*r* = 0.307) were observed. In the control group, the relationships did not reach the level of statistical significance (*p* > 0.05). The highest positive correlations with E-DII score were observed for PAI-1 (*r* = 0.160) and fasting blood glucose (*r* = 0.139), while the highest negative correlation was observed for vaspin (*r* = −0.110).

In the T2DM group, variables showing a significant negative correlation with dietary macronutrients and micronutrients based on E-DII scores and 3-day average food consumption records included fiber (*r* = −0.425; *p* = 0.004), plant-based protein (*r* = −0.370; *p* = 0.012), total fat (*r* = −0.316; *p* = 0.035), vitamin E (*r* = −0.445; *p* = 0.002), vitamin C (*r* = −0.501; *p* < 0.001), zinc (*r* = −0.473; *p* < 0.001), and magnesium (*r* = −0.628; *p* < 0.001; Table 4).

**Table 4:** Correlations (*r*) between dietary macronutrients and micronutrients according to average 3-day food consumption records and E-DII.

Variables^*^		E-DII		Variables^*^		E-DII	
		T2DM group (*n* = 45)	Control group (*n* = 45)			T2DM group (*n* = 45)	Control group (*n* = 45)
Carbohydrate (g)	*r*	−0.290	−0.262	Vitamin A (μg)	*r*	−0.167	−0.404
	*p*	0.054	0.082		*p*	0.273	**0.006**
Carbohydrate (%)	*r*	−0.058	0.090	Vitamin E (mg)	*r*	−0.445	−0.570
	*p*	0.704	0.557		*p*	**0.002**	**< 0.001**
Fiber (g)	*r*	−0.425	−0.455	Thiamine (mg)	*r*	−0.516	−0.603
	*p*	**0.004**	**0.002**		*p*	**< 0.001**	**< 0.001**
Protein (g)	*r*	−0.289	−0.464	Riboflavin (mg)	*r*	−0.221	−0.328
	*p*	0.054	**0.001**		*p*	0.144	**0.028**
Protein (%)	*r*	0.044	−0.250	Folate (μg)	*r*	−0.526	−0.665
	*p*	0.772	0.098		*p*	**< 0.001**	**< 0.001**
Plant-derived protein (g)	*r*	−0.370	−0.492	Vitamin C (mg)	*r*	−0.501	−0.489
	*p*	**0.012**	**< 0.001**		*p*	**< 0.001**	**< 0.001**
Fat (g)	*r*	−0.316	−0.466	Zinc (mg)	*r*	−0.473	−0.567
	*p*	**0.035**	**0.001**		*p*	**< 0.001**	**< 0.001**
Fat (%)	*r*	0.078	0.022	Magnesium (mg)	*r*	−0.628	−0.582
	*p*	0.611	0.886		*p*	**< 0.001**	**< 0.001**
Saturated fatty acid (g)	*r*	−0.218	−0.370	Selenium (μg)	*r*	0.064	0.217
	*p*	0.151	**0.012**		*p*	0.675	0.152
Monounsaturated acid (g)	*r*	−0.222	−0.353				
	*p*	0.143	**0.017**				
Polyunsaturated acid (g)	*r*	−0.386	−0.596				
	*p*	**0.009**	**< 0.001**				

^*^Pearson correlation coefficients were used to examine relationships between two quantitative variables for data with normal distribution; Spearman correlation coefficients were used when at least one of the variables did not have normal distribution. Values with *p* < 0.05 are written in bold.

## Discussion

This study demonstrated that women with T2DM had significantly higher PAI-1 levels compared to controls, whereas vaspin levels did not differ significantly between the groups. Furthermore, this study has shown that adiposity index values and cardiometabolic risk parameters are higher in women with T2DM. The variables most strongly associated with T2DM in this study included HbA1c, PAI-1, FRS, total cholesterol, low HDL-C levels, and high DII and E-DII scores.

The development and course of T2DM are both closely related not only to glucose metabolism disorders but also to body composition and fat distribution (19). In particular, increased BMI, abdominal obesity, and visceral fat are among the key determinants of insulin resistance and significantly increase the risk of T2DM (20). In predicting T2DM, adiposity indices (i.e., VAI, BAI, LAP, and CI) are considered more powerful predictors than classical anthropometric measurements (21). In the present study, VAI, LAP, and CI values were found to be significantly higher in the case group compared to the control group. This difference may be attributed to factors such as age, BMI, and waist circumference (22, 23). These data highlight the strong association between abdominal fat and dyslipidemia in T2DM and the importance of adiposity indices in case management.

T2DM increases the risk of coronary artery disease, stroke, and vascular mortality, and these relationships are more pronounced in women, independently of other factors (24). Cardiovascular disease increases mortality, shortening the lifespan of individuals with diabetes by approximately 10 years (25). The FRS is a widely used tool with proven effectiveness in assessing cardiovascular risk in patients with T2DM (24, 26). In this study, FRS values were found to be higher in individuals with T2DM compared to the control group. These results indicate that the FRS is a strong predictor of cardiovascular disease risk in T2DM and, when evaluated together with factors such as age, dyslipidemia, and hypertension, can influence clinical decision-making processes (27).

PAI-1 is associated with hypofibrinolysis and increased thrombotic risk in T2DM (28). PAI-1 levels have been reported to be higher in individuals with T2DM compared to control groups, and this increase is particularly pronounced in the presence of poor glycemic control (29, 30). The increased PAI-1 activity in these studies is associated with decreased fibrinolytic activity and may be influenced by factors such as hyperglycemia, insulin resistance, and age. Vaspin, on the other hand, is associated with obesity, T2DM, and metabolic syndrome and is being considered as a potential therapeutic candidate due to its anti-inflammatory and protective properties (31, 32). Serum vaspin levels range from 200 to 250 pg/mL and are affected by sex, obesity, medication use, and lifestyle factors (31, 32). The literature contains conflicting findings, with studies reporting both increased and decreased vaspin levels in obese individuals and T2DM patients, a phenomenon explained by biological variation and vaspin's complex role in pathophysiological processes (31, 33). In the present study, although mean vaspin levels were numerically higher in the T2DM group, the difference between groups was not statistically significant. The lack of a significant difference in vaspin levels between groups may be related to the relatively small sample size, biological variability of vaspin secretion, and the influence of obesity-related metabolic factors. The correlation between PAI-1 and vaspin can be explained by PAI-1's central role in inflammatory processes and insulin resistance and by vaspin's increase under stressful conditions through protective mechanisms. The combined assessment of both biomarkers is important for understanding the inflammatory and metabolic risk profile of T2DM. Given the high prevalence of T2DM and the risk of complications, early identification of risk factors is crucial for prevention strategies (34–36). Lifestyle changes have been shown to prevent the disease, making the identification of individual risk factors a priority (36, 37). Age is a non-modifiable factor (38, 39), and in this study higher age was significantly associated with T2DM status. The literature confirms this association of age with the development of diabetes and its complications (36, 40). Current predictive models primarily use biomarkers such as HbA1c, fasting glucose, and total cholesterol to measure this relationship (41–43). In the present study, higher HbA1c levels were strongly associated with higher odds of T2DM (OR: 7.828, 95% CI: 2.556–23.980, *p* < 0.001).

PAI-1 is another important biomarker that suppresses fibrinolysis and plays a role in the pathophysiology of diabetes (44, 45). In this study, each 1 ng/mL increase in PAI-1 levels was significantly associated with T2DM status, whereas vaspin levels showed a modest but significant association with T2DM status. The FRS is more often used in predicting cardiovascular risk, but it is thought to predict the development of T2DM as well (46). A study by Kwon et al. in Korea with individuals with prediabetes and T2DM showed that FRS values were higher in individuals with diabetes and that these values increased further with the progression of diabetes (46). FRS was significantly associated with higher odds of T2DM in the present study (OR: 1.117, 95% CI: 1.031–1.210, *p* = 0.007).

The inflammatory potential of the diet has been suggested to be associated with metabolic disorders, including T2DM. High DII scores reflect pro-inflammatory dietary patterns and have been linked to adverse cardiometabolic outcomes (47). The literature reports associations between DII and chronic conditions such as cardiovascular diseases, obesity, and T2DM (48, 49). For example, Denova-Gutiérrez et al. reported that individuals with higher DII scores had a greater likelihood of T2DM compared with those with lower scores (48). Similarly, a study conducted in China observed a higher prevalence of T2DM among individuals with the highest DII scores (49). In contrast to these findings, the present study did not demonstrate a statistically significant association between DII or E-DII scores and T2DM status after adjustment for potential confounders. This discrepancy may be related to differences in study design, sample characteristics, medication use, dietary assessment methods, or the relatively small sample size. Additionally, the cross-sectional nature of the study limits the ability to capture long-term dietary exposure and temporal relationships. Nevertheless, the observed non-significant trends and existing evidence suggest that the inflammatory potential of the diet may still be relevant to metabolic health and warrants further investigation.

Pro-inflammatory dietary components, particularly saturated fats and refined carbohydrates, are associated with insulin resistance and metabolic disorders, while anti-inflammatory elements like fiber can have protective effects (47). The DII is associated with abdominal obesity and fat accumulation, and high scores have been shown to correlate positively with waist circumference, body fat percentage, and visceral fat ratio (50). In individuals with T2DM, high DII scores are positively associated with cardiometabolic risk markers such as HOMA-IR score, and blood pressure (51, 52). This study similarly found significant positive correlations between E-DII and waist circumference, fat mass, arterial blood pressure, insulin, HOMA-IR, and blood pressure. This suggests that inflammatory dietary patterns may increase metabolic disruption. The absence of significant correlations in the control group may be explained by the fact that inflammatory processes are not significantly activated in healthy individuals.

Carbohydrate types are thought to play a decisive role in inflammation. Diets rich in complex carbohydrates and fiber contribute to the suppression of inflammatory markers, while excessive consumption of refined carbohydrates is associated with an increase in CRP and other inflammatory cytokines (53). In the present study, a negative correlation was observed between E-DII and fiber intake in the case group. This suggests that diets with low inflammatory potential may have higher fiber content. Furthermore, vitamins A, C, and E can modulate inflammatory processes due to their antioxidant properties. Negative associations with DII have been shown in the literature (54), and the present study also revealed negative correlations between E-DII score and both vitamin C and vitamin E intake. These findings suggest that vitamins E and C are important micronutrients that may affect the DII score, and diets with low inflammatory potential may result in greater consumption of these vitamins.

## Strengths and limitations

This study has some limitations. The inclusion of only women limits the generalizability of the results. In addition, dietary intake was assessed using a 3-day dietary record, which may be subject to reactivity bias and underreporting of energy intake. Furthermore, the examination of PAI-1 and vaspin biomarkers does not reflect all aspects of the inflammatory process. Due to the cross-sectional design, the findings do not reveal a causal relationship. Antidiabetic medication use represents an important potential confounder because such treatments may directly influence glucose metabolism, insulin sensitivity, and inflammatory biomarkers. However, because medication use was almost entirely restricted to the T2DM group and absent in controls, statistical adjustment was not possible due to quasi-complete separation. Consequently, residual confounding by treatment effects cannot be fully ruled out. However, this study also has noteworthy strengths. The unique aspect of this study is its holistic analysis of biochemical and nutritional inflammatory profiles in women with diabetes based on the evaluation of PAI-1, vaspin, and DII components together. Thus, this study provides evidence that may be relevant for understanding individual variability in dietary inflammatory load and adipokine profiles.

## Conclusions

This study has demonstrated that PAI-1 and vaspin levels are associated with increased cardiometabolic risk in women with T2DM and that these biomarkers may be linked to diet quality. Furthermore, higher PAI-1, vaspin, age, and HbA1c levels were associated with higher odds of T2DM. A positive correlation was found between the DII and anthropometric and metabolic parameters, while negative correlations were observed with some micronutrients and macronutrients. These findings highlight the impact of nutrition-induced inflammation on T2DM and related risk factors, reflecting the importance of biomarker-based approaches in individualizing nutritional therapy.

## Data Availability

The original contributions presented in the study are included in the article/supplementary material, further inquiries can be directed to the corresponding author.
